# Implementation outcomes of a VTE prevention and management system within the close-knit county medical alliance model

**DOI:** 10.3389/fpubh.2026.1802075

**Published:** 2026-03-18

**Authors:** Xiuli Zhang, Jinwang Liu, Jian Wei, Chunxian Lin, Wei Yang, Mali Wang, Lingli Huang, Bo Zheng

**Affiliations:** 1 Department of Medical, Chengdu Qingbaijiang District People's Hospital, Chengdu, China; 2 Department of Orthopaedic Surgery, The First Affiliated Hospital of Chengdu Medical College, Chengdu, China; 3 Department of Healthcare, General Hospital of Western Theater Command of the People's Liberation Army of China, Chengdu, China

**Keywords:** close-knit county medical alliance, computer-supported technology, county-level hospital, quality improvement, township hospital, VTE prevention and management

## Abstract

**Background:**

Venous thromboembolism (VTE) prevention and management has been identified as a key priority area for healthcare quality and safety in Chinese hospitals. The National Health Commission has incorporated “improving the standardized prophylaxis rate for venous thromboembolism” as one of the national medical quality and safety improvement goals for China from 2021 to 2025. While large tertiary hospitals in China have actively implemented VTE prevention and management systems with notable success, significant challenges persist at the grassroots level, including insufficient VTE prevention capabilities and low awareness in county-level and township hospitals.

**Methods:**

Inpatients from the consortium (comprising 1 county-level hospital and 6 township hospitals) between July 2023 and June 2025 were selected as the study population. July 2024 was designated as the project initiation timepoint. The period from July 2023 to June 2024 was defined as the pre-intervention phase serving as the control group (CG), while the period from July 2024 to June 2025 was defined as the post-intervention phase serving as the observation group (OG). The analysis focused on trends in changes of indicators such as in-hospital VTE detection/diagnosis rate, risk assessment rate, bleeding risk assessment rate, mechanical prophylaxis implementation rate, and pharmacological prophylaxis implementation rate before and after the initiation of the VTE prevention and management project.

**Results:**

In the county-level hospital, the observation group comprised 30,863 cases and the control group 31,382 cases. Across the six township hospitals, the observation group included 35,204 cases and the control group 34,751 cases. There were no statistically significant differences in gender, age, or body mass index between the two groups in either the county-level or township hospitals (*p* > 0.05). In the county-level hospital, the observation group showed significantly higher rates than the control group in VTE risk assessment (99.47% vs. 67.42%), in-hospital VTE detection/diagnosis (1.42% vs. 0.64%), bleeding risk assessment (79.76% vs. 46.76%), mechanical prophylaxis implementation (46.15% vs. 20.96%), and pharmacological prophylaxis implementation (48.10% vs. 21.71%), with all differences being statistically significant (*p* < 0.05). Similarly, in the township hospitals, the observation group demonstrated significantly higher rates than the control group in VTE risk assessment (98.43% vs. 23.15%), in-hospital VTE detection/diagnosis (0.55% vs. 0.26%), bleeding risk assessment (81.25% vs. 41.15%), mechanical prophylaxis implementation (29.28% vs. 14.37%), and pharmacological prophylaxis implementation (8.49% vs. 2.54%), with all differences being statistically significant (*p* < 0.05).

**Conclusion:**

Under the close-knit county medical alliance model and with the application of computer-supported technology, improvements were observed in VTE risk assessment rates, in-hospital VTE detection/diagnosis rate, bleeding risk assessment rates, mechanical prophylaxis implementation rates, and pharmacological prophylaxis implementation rates in both county-level and township hospitals.

## 1 Introduction

VTE is a serious clinical condition, representing the third most common vascular disease after acute myocardial infarction and stroke, and is characterized by high mortality and disability rates. It is also a common complication among hospitalized patients (1–3). VTE not only poses significant health risks to patients but also imposes a substantial economic burden by increasing healthcare costs, prolonging recovery time, and extending length of stay (LOS), highlighting the importance of implementing effective prevention and management systems (4, 5). Globally, the incidence of VTE is estimated to range from 115 to 269 cases per 100,000 population, with mortality rates ranging from 6.8 to 32.3 per 100,000 population. VTE consumes considerable healthcare resources, with its annual cost in the United States estimated to be between $13.5 billion and $27.2 billion (4–7). In China, the prevalence of VTE has increased from 3.2 per 100,000 population in 2007 to 17.5 per 100,000 population in 2016 (8).

Despite significant advancements in the prevention and treatment of VTE over the past few decades, numerous challenges persist in clinical practice in China, including insufficient implementation rates of risk assessment and preventive measures, with standardized VTE prophylaxis rates remaining below international levels (9). Recognizing VTE prevention and management as a critical component of hospital healthcare quality and safety, the National Health Commission has incorporated “improving the standardized prophylaxis rate for venous thromboembolism” into the national medical quality and safety improvement goals for 2021–2025. To standardize the clinical management of VTE in China, the VTE Prevention and Management Capacity Building Project was officially launched in 2018. An expert committee was established to develop the *Guidelines for Quality Evaluation and Management of In-Hospital Venous Thromboembolism Prevention and Treatment*, aiming to establish VTE prevention and management systems within hospitals at all levels and reduce the incidence of fatal VTE cases. While favorable national policies have encouraged many large hospitals in China to actively implement VTE prevention and management, the capacity for VTE prevention and management in county-level and township hospitals remains inadequate (9, 10).

Globally, efforts have been made to implement in-hospital thromboprophylaxis, with scientific bodies updating VTE prevention guidelines (11). Notably, several international hospitals have successfully formed evidence-based VTE prevention teams. For instance, Johns Hopkins Hospital in the U.S. created a multidisciplinary team to develop a standardized VTE prevention process using a clinical decision support system, enhancing risk assessment and prophylaxis rates (12).

The county medical alliance, also known as the county-level integrated healthcare delivery system, represents a collaborative framework in which medical groups serve as one of its key operational forms. First proposed in 2017, this model refers to a three-tiered, interconnected healthcare service system comprising county-level hospitals, township health centers, and village clinics (9, 10, 13). The medical alliance model facilitates rational patient triage, promotes the sharing of high-quality medical resources, and advances the equitable allocation of healthcare resources, thereby enhancing overall service quality (14, 15).

To enhance the capacity for VTE prevention and management in county-level and township hospitals, this study employed a observational study design within a close-knit county medical alliance framework, utilizing information technology to evaluate the effectiveness of VTE prevention and management compared to traditional approaches. This study aims to leverage the close-knit medical alliance model to improve VTE prevention and management capabilities in county-level and township hospitals. Furthermore, it seeks to explore its potential contribution to reducing in-hospital VTE detection/diagnosis rate and improving patient prognosis, thereby offering guidance for future clinical practice.

## 2 Methods

### 2.1 Study design

**Figure 1:**
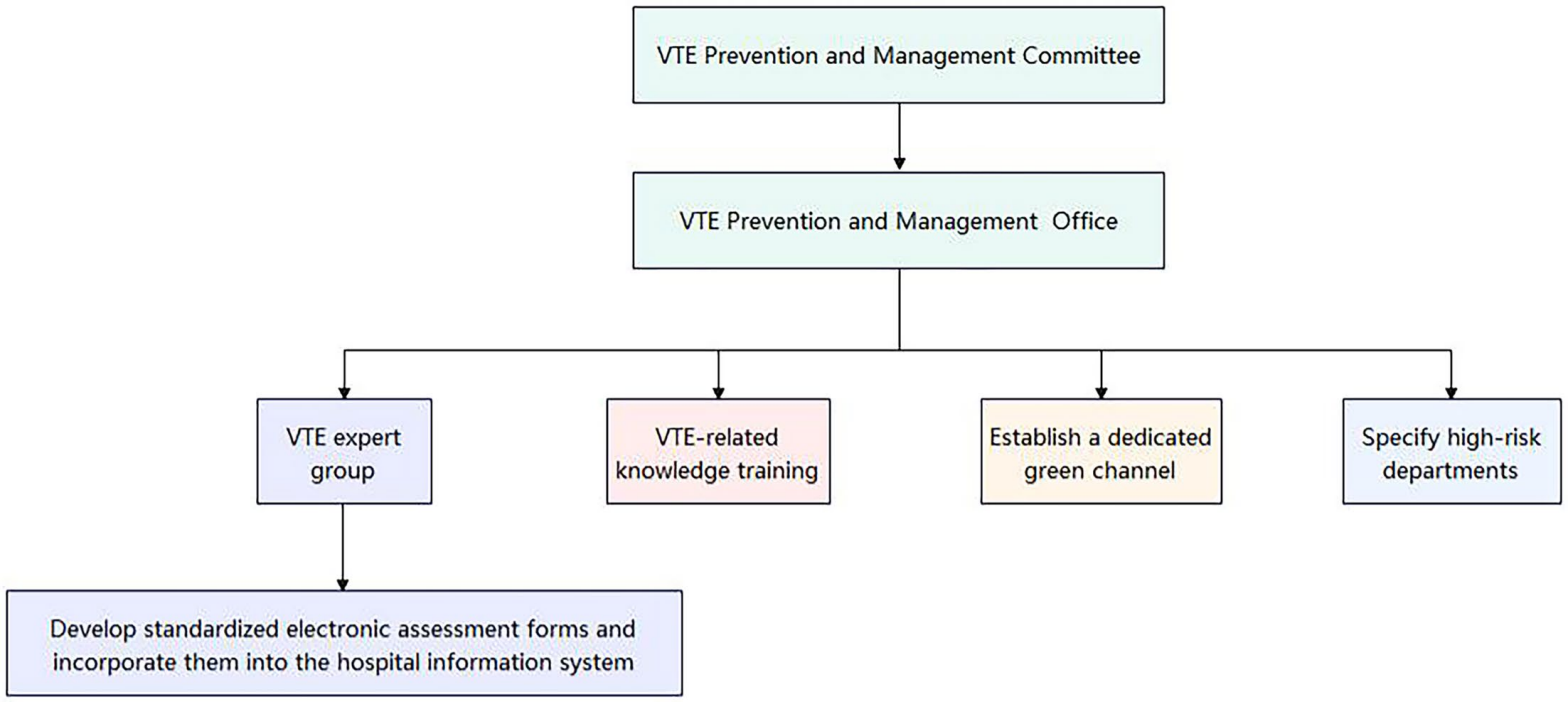
Organizational structure of the VTE prevention and management system.

**Figure 2:**
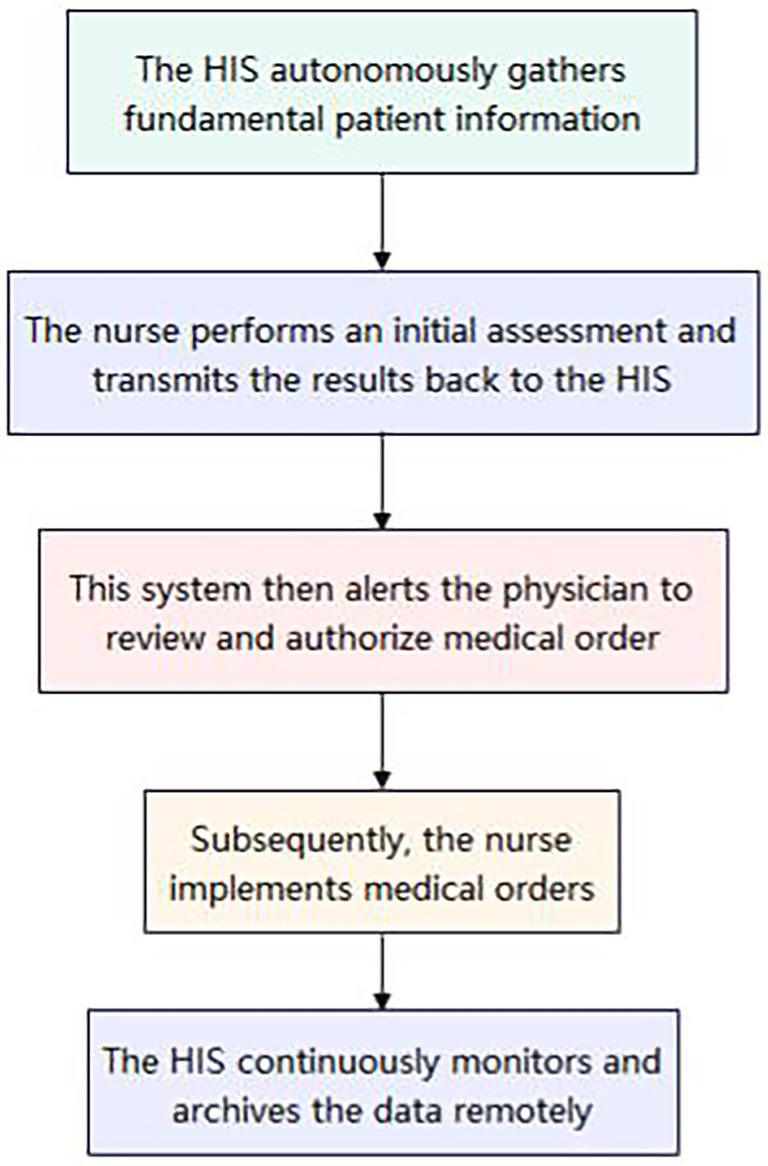
Prevention and treatment process for VTE in hospitalized patients. HIS, Hospital Information System.

This observational study was conducted from July 2023 to June 2025 in Qingbaijiang District, Chengdu, Sichuan Province, China. The consortium was led by a county-level hospital, with six township health centers as member units. The study population consisted of hospitalized patients within the consortium. Beginning in July 2024, a comprehensive system for the prevention and management of venous thromboembolism (VTE) was implemented across the consortium. This initiative included the formation of a unified VTE Prevention and Management Committee and Office, the establishment of a VTE Expert Group, the organization of VTE-related knowledge training programs, the creation of a dedicated green channel, and the identification of high-risk departments (Figure 1). The expert group developed standardized electronic assessment forms integrated into the hospital information system (HIS) (using the Caprini score for surgical patients and the Padua score for non-surgical patients). The HIS autonomously gathers fundamental patient information. Subsequently, the nurse performs an initial assessment and transmits the results back to the HIS. This system then alerts the physician to review and authorize medical orders, which the nurse subsequently implements. Additionally, the HIS continuously monitors and archives the data remotely (Figure 2). For patients at moderate-to-high risk of thrombosis, physicians conducted bleeding risk assessments and evaluated contraindications for prophylaxis, then prescribed preventive measures (mechanical and/or pharmacological prophylaxis). Nurses executed the prescribed orders. For patients with suspected VTE, a green channel is immediately activated to facilitate expert consultation, referral, and treatment, along with the implementation of relevant measures, while regular continuous quality improvement is carried out.

Therefore, this study defined the period from July 2024 to June 2025 as the post-intervention phase (observation group, OG), and the period from July 2023 to June 2024 was designated as the pre-intervention phase, serving as the control group (CG), during which the seven hospitals implemented their respective customized VTE intervention protocols, including paper-based thrombosis risk assessment and bleeding risk assessment, followed by corresponding interventions. The study was approved by the Ethics Committee of Qingbaijiang District People’s Hospital (Approval No. 202527) and conducted in accordance with the Declaration of Helsinki. Written informed consent was obtained from all participants.

Inclusion criteria: ① Hospital stay > 24 h; ② Age > 14 years; ③ No history of PE or DVT prior to admission. Exclusion criteria: ① Outpatients or emergency department patients; ② Day-surgery procedure patients; ③ Hospitalized patients in neonatology or pediatrics departments.

### 2.2 Evaluation methods

Age, gender, and body mass index were compared between the two groups. The primary and secondary outcomes were also compared. The primary outcomes included: VTE risk assessment (%) (Total number of discharged patients across all categories who received VTE risk assessment at different dynamic time points/Total number of discharged patients across all categories during the same period); bleeding risk assessment (%) (Total number of discharged patients across all categories who met the denominator criteria and underwent bleeding risk assessment within the corresponding dynamic time points/Total number of discharged patients across all categories whose VTE risk assessment results indicated moderate-to-high risk across different dynamic time points); adoption of mechanical prophylaxis (%) (Total number of discharged patients across all categories who met the denominator criteria and were prescribed mechanical prophylaxis orders within the corresponding dynamic time points/Total number of discharged patients across all categories whose VTE risk assessment results indicated moderate-to-high risk across different dynamic time points); adoption of pharmacological prophylaxis (%) (Total number of discharged patients across all categories who met the denominator criteria and were prescribed pharmacological prophylaxis orders within the corresponding dynamic time points/Total number of discharged patients across all categories who were assessed as having moderate-to-high VTE risk and low bleeding risk across different dynamic time points); in-hospital VTE detection/diagnosis (%) (Total number of patients discharged with hospital-confirmed VTE/Total number of patients discharged during the same period). The secondary outcomes included: Moderate-to-high VTE risk (%) (Number of discharged patients with at least one VTE risk assessment conducted during hospitalization indicating moderate-to-high risk/Total number of discharged patients who underwent VTE risk assessment during the same period); high risk of bleeding (%) (Number of discharged patients with at least one bleeding risk assessment conducted during hospitalization indicating high risk/Total number of discharged patients who underwent bleeding risk assessment(s) during the same period).

### 2.3 Statistical methods

In this study, two distinct groups were established: the observation group (OG) and the control group (CG). The independent variable served as the grouping variable, while the dependent variable represented the outcome or observed variable. Data analysis was performed using SPSS software, version 21.0. Descriptive statistics for continuous data were expressed as mean ± standard deviation, and categorical data were presented as percentages. When sample data followed a normal distribution, the Student’s t-test was employed for comparisons between the two groups; otherwise, the bootstrap Welch t-test was applied. If excessive outliers were present, the Yuen–Welch approach was used. The chi-square test was utilized to assess differences in frequency distributions between the two groups. For repeated-measures data, analysis of variance (ANOVA) was conducted if the data were normally distributed; otherwise, robust ANOVA was employed. A significance level of *p* < 0.05 was set for all statistical analyses.

## 3 Results

In the county-level tertiary hospital, the observation group comprised 30,863 patient cases and the control group 31,382 patient cases. Across the six township hospitals, the observation group included 35,204 patient cases and the control group 34,751 patient cases. There were no statistically significant differences in gender, age, or body mass index (BMI) between the two groups in either the county-level tertiary hospital or the township hospitals (*p* > 0.05, Tables 1, 2).

**Table 1:** General characteristics of the two groups in one county-level hospital.

Variables	OG (*n* = 30,863)	CG (*n* = 31,382)	*t*/*χ*^2^	*p*
Gender			2.08	0.14
Man	14,253	14,674		
Female	16,610	16,708		
Age (year)	65.05 ± 5.11	64.76 ± 2.13	1.24	1.41
BMI (kg/m^2^)	21.07 ± 1.21	21.32 ± 0.85	0.54	0.86

OG, the observation group (from July 2024 to June 2025); CG, the control group (from July 2023 to June 2024); BMI, body mass index.

**Table 2:** General characteristics of the township hospitals.

Variables	OG (*n* = 35,204)	CG (*n* = 34,751)	*t*/*χ*^2^	*p*
Gender			0.03	0.85
Man	16,119	15,935		
Female	19,085	18,816		
Age (year)	61.24 ± 1.06	61.81 ± 0.59	0.48	0.52
BMI (kg/m^2^)	21.52 ± 1.68	21.11 ± 0.95	1.55	1.71

OG, the observation group (from July 2024 to June 2025); CG, the control group (from July 2023 to June 2024); BMI, body mass index.

**Figure 3:**
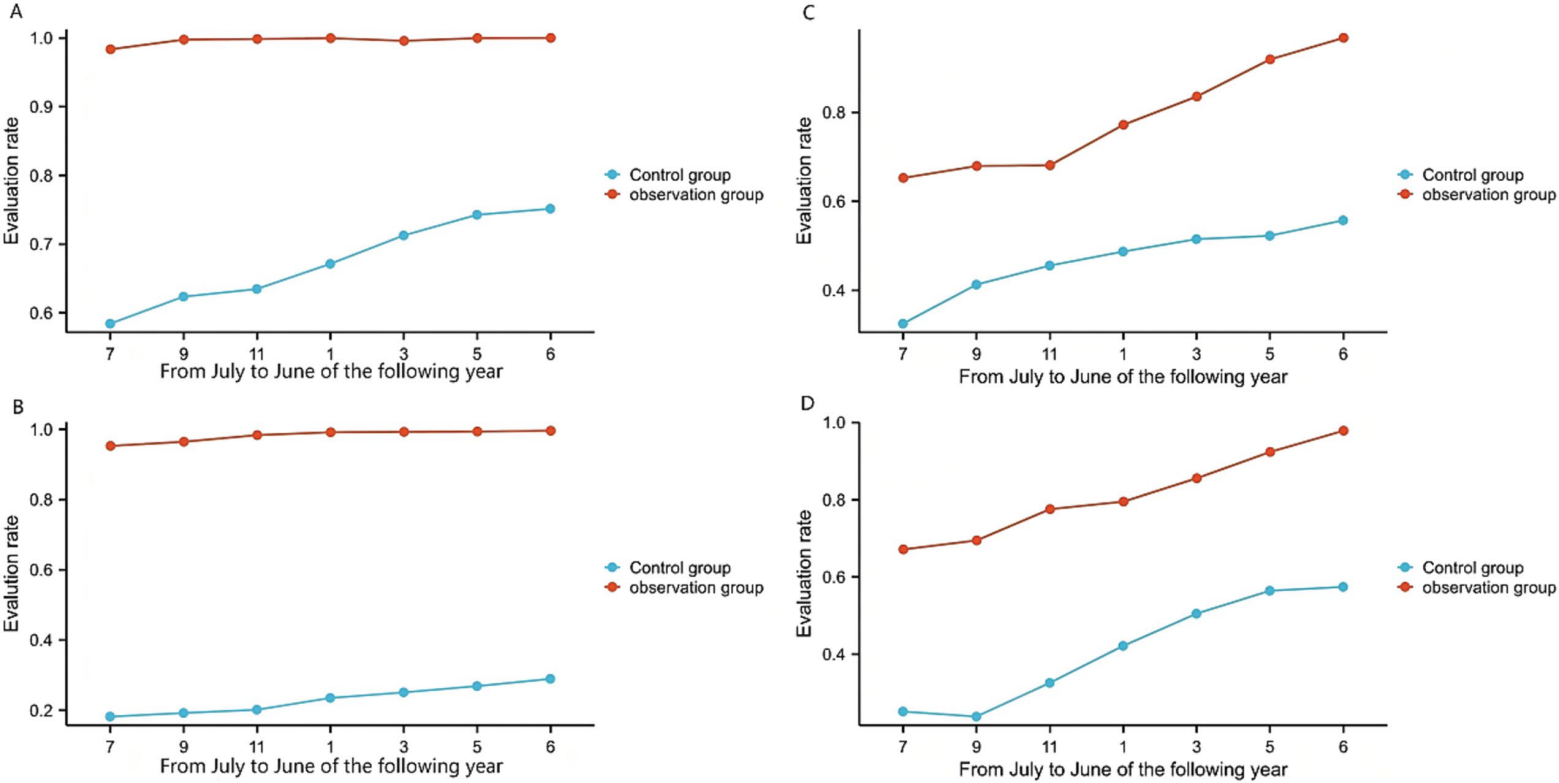
Assessment rates. **(A)** The VTE risk assessment rate of a county-level tertiary hospital. **(B)** The VTE risk assessment rate of 6 township hospitals. **(C)** The rate of bleeding risk assessment of a county-level tertiary hospital. **(D)** The rate of bleeding risk assessment of 6 township hospitals.

**Figure 4:**
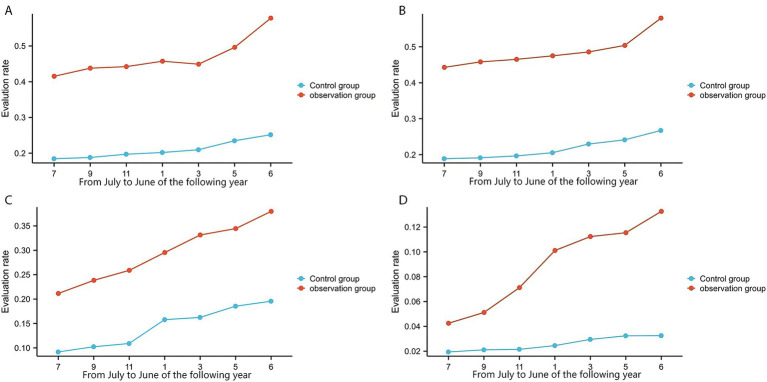
Prophylaxis implementation rates. **(A)** The mechanical prophylaxis adoption rate of a county-level tertiary hospital. **(B)** The mechanical prophylaxis adoption rate of 6 township hospitals. **(C)** The pharmacological prophylaxis adoption rate of a county-level tertiary hospital. **(D)** The pharmacological prophylaxis adoption rate of 6 township hospitals.

**Figure 5:**
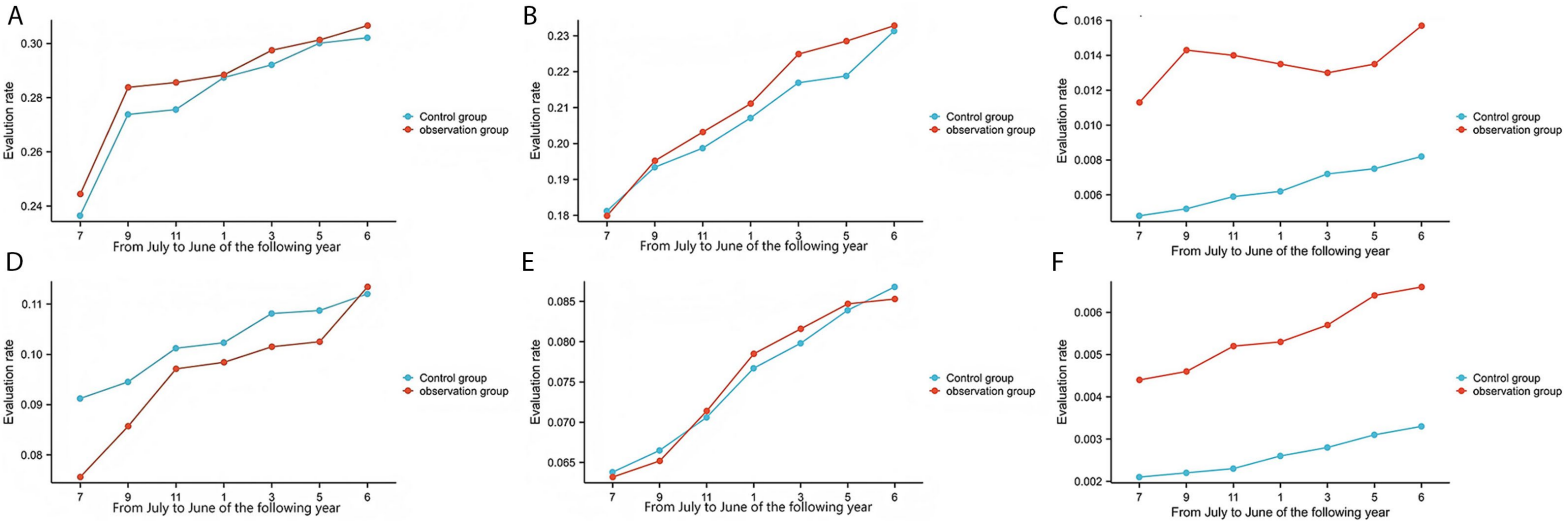
Event rates. **(A)** The moderate-to-high VTE risk rate of a county-level tertiary hospital. **(B)** The moderate-to-high VTE risk rate of 6 township hospitals. **(C)** The high bleeding risk rate of a county-level tertiary hospital. **(D)** The high bleeding risk rate of 6 township hospitals. **(E)** The in-hospital VTE detection/diagnosis rate of a county-level tertiary hospital. **(F)** The in-hospital VTE detection/diagnosis rate of 6 township hospitals.

In the county-level tertiary hospital, the observation group and the control group comprised 30,863 and 31,382 patient cases, respectively. The observation group demonstrated significantly higher rates than the control group in VTE risk assessment (99.47% vs. 67.42%, Figure 3), bleeding risk assessment (79.76% vs. 46.76%, Figure 3), adoption of mechanical prophylaxis (46.15% vs. 20.96%, Figure 4), adoption of pharmacological prophylaxis (48.10% vs. 21.71%, Figure 4), and in-hospital VTE detection/diagnosis (1.42% vs. 0.64%, Figure 5), with all differences being statistically significant (*p* < 0.05, Table 3). There were no statistically significant differences between the two groups in the proportion of patients at moderate-to-high risk of VTE (28.80% vs. 28.28%, Figure 5) or the proportion at high risk of bleeding (20.21% vs. 20.69%, Figure 5) (*p* > 0.05, Table 3).

**Table 3:** The primary and secondary outcomes for a county-level tertiary hospital.

Variables	OG (*n* = 30,863)	CG (*n* = 31,382)	*χ* ^2^	*p*
① VTE risk assessment (%)	99.47% (30,706)	67.42% (21,157)	11518.12	0.00
② Bleeding risk assessment (%)	79.76% (7055/8845)	46.76% (2798/5984)	369.17	0.00
③ Adoption of mechanical prophylaxis (%)	46.15% (4082/8845)	20.96% (1254/5984)	484.33	0.00
④ Adoption of pharmacological prophylaxis (%)	48.10% (2816/5854)	21.71% (654/3012)	273.16	0.00
⑤ Moderate-to-high VTE risk (%)	28.80% (8845/30706)	28.28% (5984/21157)	0.92	0.33
⑥ High risk of bleeding (%)	20.21% (1362/6737)	20.69% (579/2798)	0.183	0.66
⑦ In-hospital VTE detection/diagnosis (%)	1.42% (439)	0.64% (201)	93.48	0.00

OG, the observation group (from July 2024 to June 2025); CG, the control group (from July 2023 to June 2024); ① VTE risk assessment (%) (Total number of discharged patients across all categories who received VTE risk assessment at different dynamic time points/Total number of discharged patients across all categories during the same period); ② bleeding risk assessment (%) (Total number of discharged patients across all categories who met the denominator criteria and underwent bleeding risk assessment within the corresponding dynamic time points/Total number of discharged patients across all categories whose VTE risk assessment results indicated moderate-to-high risk across different dynamic time points); ③ adoption of mechanical prophylaxis (%) (Total number of discharged patients across all categories who met the denominator criteria and were prescribed mechanical prophylaxis orders within the corresponding dynamic time points/Total number of discharged patients across all categories whose VTE risk assessment results indicated moderate-to-high risk across different dynamic time points); ④ adoption of pharmacological prophylaxis (%) (Total number of discharged patients across all categories who met the denominator criteria and were prescribed pharmacological prophylaxis orders within the corresponding dynamic time points/Total number of discharged patients across all categories who were assessed as having moderate-to-high VTE risk and low bleeding risk across different dynamic time points); ⑤ moderate-to-high VTE risk (%) (Number of discharged patients with at least one VTE risk assessment conducted during hospitalization indicating moderate-to-high risk/Total number of discharged patients who underwent VTE risk assessment during the same period); ⑥ high risk of bleeding (%) (Number of discharged patients with at least one bleeding risk assessment conducted during hospitalization indicating high risk/Total number of discharged patients who underwent bleeding risk assessment(s) during the same period); ⑦ in-hospital VTE detection/diagnosis (%) (Total number of patients discharged with hospital-confirmed VTE/Total number of patients discharged during the same period).

In the township hospitals, the observation group and the control group comprised 35,204 and 34,751 patient cases, respectively. The observation group demonstrated significantly higher rates than the control group in VTE risk assessment (98.43% vs. 23.15%, Figure 3), bleeding risk assessment (81.25% vs. 41.15%, Figure 3), adoption of mechanical prophylaxis (29.28% vs. 14.37%, Figure 4), adoption of pharmacological prophylaxis (8.49% vs. 2.54%, Figure 4), and in-hospital VTE detection/diagnosis (0.55% vs. 0.26%, Figure 5), with all differences being statistically significant (*p* < 0.05, Table 4). There were no statistically significant differences between the two groups in the proportion of patients at moderate-to-high VTE risk (9.67% vs. 10.12%, Figure 5) or the proportion at high risk of bleeding (7.42% vs. 7.76%, Figure 5) (*p* > 0.05, Table 4).

**Table 4:** The primary and secondary outcomes for 6 township hospitals.

Variables	OG (*n* = 35,204)	CG (*n* = 34,751)	*t*/*χ*^2^	*p*
① VTE risk assessment (%)	98.43% (34,654)	23.15% (8,045)	41681.30	0.00
② Bleeding risk assessment (%)	81.25% (2723/3350)	41.15% (335/814)	97.32	0.00
③ Adoption of mechanical prophylaxis (%)	29.28% (981/3350)	14.37% (117/814)	47.18	0.00
④ Adoption of pharmacological prophylaxis (%)	8.49% (219/2587)	2.54% (25/983)	35.11	0.00
⑤ Moderate-to-high VTE risk (%)	9.67% (3350/34654)	10.12% (814/8045)	1.23	0.26
⑥ High risk of bleeding (%)	7.42% (202/2723)	7.76% (25/335)	0.001	0.97
⑦ In-hospital VTE detection/diagnosis (%)	0.55% (192)	0.26% (90)	35.73	0.00

OG, the observation group (from July 2024 to June 2025); CG, the control group (from July 2023 to June 2024); ① VTE risk assessment (%) (Total number of discharged patients across all categories who received VTE risk assessment at different dynamic time points/Total number of discharged patients across all categories during the same period); ② bleeding risk assessment (%) (Total number of discharged patients across all categories who met the denominator criteria and underwent bleeding risk assessment within the corresponding dynamic time points/Total number of discharged patients across all categories whose VTE risk assessment results indicated moderate-to-high risk across different dynamic time points); ③ adoption of mechanical prophylaxis (%) (Total number of discharged patients across all categories who met the denominator criteria and were prescribed mechanical prophylaxis orders within the corresponding dynamic time points/Total number of discharged patients across all categories whose VTE risk assessment results indicated moderate-to-high risk across different dynamic time points); ④ adoption of pharmacological prophylaxis (%) (Total number of discharged patients across all categories who met the denominator criteria and were prescribed pharmacological prophylaxis orders within the corresponding dynamic time points/Total number of discharged patients across all categories who were assessed as having moderate-to-high VTE risk and low bleeding risk across different dynamic time points); ⑤ moderate-to-high VTE risk (%) (Number of discharged patients with at least one VTE risk assessment conducted during hospitalization indicating moderate-to-high risk/Total number of discharged patients who underwent VTE risk assessment during the same period); ⑥ high risk of bleeding (%) (Number of discharged patients with at least one bleeding risk assessment conducted during hospitalization indicating high risk/Total number of discharged patients who underwent bleeding risk assessment(s) during the same period); ⑦ in-hospital VTE detection/diagnosis (%) (Total number of patients discharged with hospital-confirmed VTE/Total number of patients discharged during the same period).

## 4 Discussion

Venous thromboembolism (VTE) is a serious clinical condition primarily comprising deep vein thrombosis (DVT) and pulmonary embolism (PE). Its incidence has been increasing worldwide, making it one of the leading causes of mortality among hospitalized patients (1, 2). The occurrence of VTE not only poses a serious threat to patients’ health but also imposes a substantial economic burden on healthcare systems, directly elevating healthcare expenditures and prolonging patient recovery time. While multiple domestic and international guidelines have emphasized the importance of VTE prevention and management, the implementation of thromboprophylaxis in clinical practice remains suboptimal (16, 17). A report by the American Heart Association estimates that approximately 1.22 million cases of venous thromboembolism (VTE) occur annually in the United States. This estimate is based on previously unpublished data from the Nationwide Inpatient Sample, indicating around 370,000 cases of pulmonary embolism and approximately 857,000 cases of deep vein thrombosis in 2016. Among U. S. adults, the lifetime risk of VTE is estimated to be 8%, with about 20% of individuals dying within 1 year of a VTE diagnosis—sometimes due to VTE itself, but more often due to the conditions that provoked the VTE (18–20). In China, the prevalence of VTE increased from 3.2 per 100,000 population in 2007 to 17.5 per 100,000 population in 2016 (8). Therefore, early prevention and effective management of VTE are of critical importance. Multiple studies have demonstrated that rational risk assessment and preventive measures can significantly reduce the incidence of VTE (21–23).

VTE, characterized by its high incidence and mortality rates, has garnered attention from healthcare professionals, patients, and policymakers. Worldwide, initiatives have been undertaken to implement in-hospital thromboprophylaxis, with scientific organizations issuing and updating guidelines for VTE prevention (11). Several renowned hospitals abroad have successfully established in-hospital VTE prevention and management teams based on evidence-based medicine, achieving notable outcomes. As early as 2005, the Johns Hopkins Hospital in the United States formed a multidisciplinary team comprising clinical experts, pharmacy specialists, health information technology professionals, and hospital administrators. This team established a risk assessment and intervention system based on a clinical decision support information system, which standardized the VTE prevention process and improved in-hospital VTE risk assessment and prophylaxis rates (12). In 2010, the Harborview Medical Center in Seattle established a more comprehensive interdisciplinary VTE expert committee to reduce hospital-associated VTE events. Through this model of cross-team collaboration, quality improvement, and transparent data sharing, VTE prevention measures were enhanced, thereby ensuring the safety of hospitalized patients (24). With the deepening of research on VTE prevention and management teams abroad, the development of in-hospital VTE prevention and management has gradually gained attention in China. Several large tertiary hospitals have established VTE management teams, yet implementation remains insufficient (25). Hospital-associated venous thromboembolism is a major adverse event among hospitalized patients; however, it can be prevented through standardized and appropriate prophylactic measures, including anticoagulation and mechanical interventions (15). Despite these recommendations, many hospitalized patients do not undergo risk assessment or receive appropriate prophylactic prescriptions (26). Clinical staff and hospital administrators should enhance awareness and adopt practical measures to address the growing burden of VTE (27).

Recognizing VTE prevention and management as a critical area for hospital healthcare quality and safety, the National Health Commission of China has included “improving the standardized prophylaxis rate for venous thromboembolism” as one of the national medical quality and safety improvement goals for the period 2021–2025. The county medical alliance was first proposed in 2017, referring to a three-tiered, interconnected healthcare service system comprising county-level hospitals, township health centers, and village clinics (11). To further advance the Healthy China initiative, the National Health Commission, in collaboration with the National Administration of Traditional Chinese Medicine, issued the *Notice on Promoting the Construction of Close-knit County Medical Alliances* in 2019. This measure aims to drive the development of county medical alliances and provides a guiding framework for redirecting high-quality medical resources to grassroots levels and enhancing service quality (12). Currently, China’s healthcare reform has entered a critical phase, with grassroots medical reform being a top priority. The proposal of the county medical alliance represents a significant step toward overcoming bottlenecks in primary healthcare reform. Through the construction of close-knit county medical alliances, healthcare resources within counties can be further integrated, promoting the functional positioning and strengthening collaborative division of labor among healthcare institutions at all levels. This is conducive to establishing a comprehensive, continuous, high-quality, and efficient healthcare service system that spans the entire resident health (28, 29).

Currently, the construction of county medical alliances in China has achieved certain outcomes and is advancing comprehensively. However, systematic VTE prevention and management have yet to be implemented within domestic county medical alliances, leading to insufficient capacity, limited experience, and low awareness of thrombosis prevention and management in primary healthcare institutions. The county medical alliance, also known as the county-level integrated healthcare delivery system, is operationalized in forms such as medical groups. As one of the first pilot units for medical groups in Sichuan Province, our region has adhered to a people-centered health approach. By coordinating, integrating, conserving, and redirecting resources, we have optimized the allocation of regional healthcare resources and promoted a shift in healthcare work from a “treatment-centered” to a “health-centered” model. Leveraging the implementation of close-knit county medical alliances in our region, this study aims to establish a VTE prevention and management system through the medical alliance model, paving the way for thrombosis prevention and management in our area and alleviating the burdens of thrombotic diseases for the regional population (30, 31).

This study aimed to evaluate the establishment and implementation outcomes of a VTE prevention and management system in primary county-level and township hospitals. A observational study design was employed to compare the effectiveness of VTE prevention and management between the close-knit county medical alliance model and the traditional model. The results demonstrated that the implementation of the close-knit medical alliance VTE prevention and management system significantly improved VTE risk assessment rates, in-hospital VTE detection/diagnosis rates, bleeding risk assessment rates, mechanical prophylaxis implementation rates, and pharmacological prophylaxis implementation rates in both county-level and township hospitals compared to the pre-intervention period, with all improvements being statistically significant (*p* < 0.05).

The innovation of this study lies in the systematic evaluation of the implementation outcomes of a venous thromboembolism (VTE) prevention and management system in primary county-level and township hospitals. Compared with previous research, we are the first to demonstrate that standardized VTE risk assessment and prophylactic measures can significantly improve relevant implementation rates, offering a novel perspective for VTE management. The literature indicates that the incidence of VTE is closely associated with the implementation of preventive measures (32–34), and our findings further corroborate this point, underscoring the importance of systematic management in clinical practice.

Nurses were crucial in implementing a closed-loop management process involving nurses’ initial assessments, system prompts for doctors’ review, doctors issuing orders, and nurses executing them. This approach is based on process rationality and evidence-based support, as recommended by the American Society of Hematology for individualized VTE prevention (35). Studies show that integrating structured risk assessment tools (such as Padua, Caprini scores) into electronic medical records and having trained nurses conduct initial data collection improves assessment efficiency and accuracy. Nurses, as primary bedside caregivers, are well-positioned to observe dynamic patient indicators, enhancing objective assessments (35, 36). This process follows the “assessment-decision-execution” separation principle. Nurses gather standardized information and perform initial scoring, while the system sends results to physicians for review. Physicians then confirm risk levels and issue personalized medical orders, taking full responsibility for medical decisions. This approach aligns with the multi-disciplinary collaboration model in critical care, and studies indicate it does not raise patient safety incident risks (37). Our hospital ensures nurses and doctors involved in assessments are well-prepared through: ① Pre-job specialized training on VTE guidelines, scoring tools, and case drills; ② Commencing work post internal training; ③ Ongoing refresher training and quality feedback. This aligns with institutional, national, and nursing standards, optimizing human resources by combining nurses’ observational skills with physicians’ decision-making, thus improving the timeliness and standardization of VTE prevention and ensuring medical safety (25, 38).

The findings of this study hold significant implications for clinical practice. The observed significant improvements in VTE risk assessment and prophylaxis implementation rates under the close-knit county medical alliance model indicate that primary hospitals can manage high-risk patients more effectively in daily clinical work. This enhanced management capability is expected to reduce VTE-related complications and mortality. In alignment with the analysis by Rosenow (39), effective VTE management not only improves patient prognosis but also reduces overall healthcare costs. Therefore, this study provides empirical evidence for formulating more effective VTE prevention and management policies in primary hospitals. It contributes to the advancement of clinical practice at the grassroots level and helps ensure patient safety and health.

This study demonstrates that the rate of drug prophylaxis in township hospitals (8.49%) following the intervention was significantly lower than that observed in county hospitals (48.10%). This disparity aligns with the widespread challenges encountered in implementing venous thromboembolism (VTE) prevention strategies in primary healthcare settings globally (25). The primary reasons for this discrepancy were analyzed as follows: (1) Insufficient resources and systemic support: Township hospitals often lack specialized medical personnel for VTE management, while county hospitals face inadequate staffing for comprehensive guidance, leading to a disconnect between risk assessment and preventive decision-making (38). (2) Clinical cognition and behavioral inertia: Medical staff exhibit excessive concern regarding bleeding risks, experience uncertainty in selecting guideline-recommended medications, and are influenced by entrenched practices, all of which significantly impede the initiation of preventive measures. (3) Challenges in Drug Accessibility and Implementation. Township hospitals face significant challenges due to inadequate drug inventories. Furthermore, variations in patient case profiles, particularly the high complexity of comorbidities, can further complicate assessments. The limited adaptability of existing systems to real-world grassroots scenarios also contributes to these challenges (40). (4) Deficiency in Compliance. The shortage of VTE management professionals in township hospitals impedes the provision of comprehensive explanations to patients, thereby contributing to poor patient compliance (40). In conclusion, increased financial support and the training of additional VTE management professionals are essential to address these issues effectively.

However, this study has certain limitations. First, although the sample size is substantial, the research was conducted in a specific region, which may introduce regional bias to the findings. Second, Hospitalization is defined as staying in a hospital for over 24 h. Due to bed shortages, patients waiting for beds in the emergency department were not promptly assessed or included in the study, which is a limitation. Future studies should consider multicenter trials in broader grassroots areas to further validate our results and to assess the sustained effectiveness of the close-knit county medical alliance VTE management model, as well as its impact on long-term patient outcomes (41, 42).

## 5 Conclusion

In summary, this study compared the effectiveness of VTE prevention and management between the close-knit county medical alliance model and the traditional model in primary county-level and township hospitals. The results demonstrated significant improvements in VTE risk assessment rates, in-hospital VTE detection/diagnosis rate, bleeding risk assessment rates, implementation rates of mechanical prophylaxis, and implementation rates of pharmacological prophylaxis in both county-level and township hospitals. These findings provide practical evidence for future VTE management at the primary care level and highlight the importance of systematic management in clinical application. Furthermore, the outcomes of this study offer recommendations for optimizing VTE prevention and management strategies within primary hospitals, showcasing its positive role in enhancing clinical diagnosis and treatment standards and improving patients’ quality of life.

## Data Availability

The original contributions presented in the study are included in the article/supplementary material, further inquiries can be directed to the corresponding author.
